# A Case of Glanders in the Human Subject

**Published:** 1877-07

**Authors:** Wm. Jones


A Case of Glanders in the Human Subject.—*By Dr. Wm. Jones.*—The recent death of a man from glanders or farcy (identical infections) ought to impress the people with the necessity of giving a wide berth to horses infected with that disease; and the inefficiency of all therapeutical means suggests the expediency of efficacious prophylactic measures for the compulsory destruction of the infected animals. The occult and insiduous nature of the disease, especially the chronic variety, and the difficulty of diagnosis in the incipient and early stages of its progress, attach peculiar importance and value to every pathognomonic symptom.

I was called on the fifteenth instant to see Martin Egan, aged 36 years, residing on Fifteenth street, occupation in a flour mill, subject to slight bronchial affection from inhalation of dust, but always healthy until the middle of last February, when he was taken ill with fever and rheumatism. For this he was treated until he was seized with diphtheria as we supposed, a few days before the fifteenth, previous to which no suspicion of the nature of the malady had occurred to any one, his wife and children holding uninterrupted intercourse—the latter frequently playing with and kissing him until his throat got sore.

I was informed that he had severe pains in the joints and muscles, and that lumps or bunches were noticed in the middle of the extensor muscles of the thighs and arms, but that they had disappeared; that the pains had ceased; that he was considered convalescent, being able to sit up and walk round a little. His appearance denoted extreme cachexia; his muscles were exhausted and flaccid, and the slightest attempt at voluntary motion threw them into uncontrollable agitation and tremor. There was constant subsultus tendinum. No symptoms of abscesses anywhere. Erysipelatious inflammation extended over the bridge of the nose to the malar bones, with infiltration and swelling, especially on the right side. The nares were completely filled with a foul, crusty deposit, the partial removal of which was followed by discharge of a yellowish, curdy, tuberculous matter. The lips were covered with dry blisters and the gums with a deposit of a sooty color; the tongue with a thick, dry, brown fur. The mucous membrane of the mouth and fauces was dry and parched; in some parts corroded, in others, especially on the roof of mouth and isthmus of the fauces, covered with a ragged, grayish colored coating. But the most remarkable of all was the appearance of the soft palate, which was in a dry, gangrenous condition, as if the actual cautery had just been applied, the surrounding parts being of livid red. There was neither sanious discharge, secretions, or appearance of sloughing. Deglutition was but slightly impeded. Slight bronchial rales were heard over the lungs, which were sound with that exception. The temperature was normal, his respirations 25 per minute, his pulse feeble and 130. There was decided somnolency, though his mental faculties were unimpaired. The sub-maxillary glands about the size of peas, hard as bone and painful on pressure. There was slight enlargement, but evident induration of the glands of the lymphatic system everywhere; no eruptions on the body, but in various places over the shoulders, chest and extremities, I distinctly felt round nodules about the size of small buttons, apparently rooted in the subcutaneous cellular tissue. The skin covering them became afterwards livid and putrescent.

On the 19th his pulse was not discernible. The cartilaginous portion of the nose was completely gangrened. He lapsed into a comatose condition in which he lingered for three days, until death released him on the 22d. The foregoing symptoms convinced me at first that it was not a case of diphtheria or ordinary pyemia, and enquiry as to the possibility of other infections disclosed the fact that he had doctored his horses for glanders or farcy during the winter months, and a short time before he was taken sick in February.—*Pacific Medical and Surgical Journal.*

